# Urotensin II Protects Cardiomyocytes from Apoptosis Induced by Oxidative Stress through the CSE/H_2_S Pathway

**DOI:** 10.3390/ijms160612482

**Published:** 2015-06-03

**Authors:** Hui Gong, Zhidan Chen, Xiaoyi Zhang, Yang Li, Jie Zhang, Ying Chen, Yingjiong Ding, Guoping Zhang, Chunjie Yang, Yichun Zhu, Yunzeng Zou

**Affiliations:** 1 Shanghai Institute of Cardiovascular Diseases, Zhongshan Hospital and Institutes of Biomedical Sciences, Fudan University, Shanghai 200032, China; E-Mails: ghui1975@163.com (H.G.); chenzhidan0530@163.com (Z.C.); Zhang.xiaoyi@zs-hospital.sh.cn (X.Z.); liyang9_28@163.com (Y.L.); gpzhang@fudan.edu.cn (G.Z.); yang.chunjie@zs-hospital.sh.cn (C.Y.); 2 Department of Radiology, Shanghai First People’s Hospital, School of Medicine, Shanghai Jiaotong University, Shanghai 200080, China; 3 Department of Geriatrics, Zhongshan Hospital, Fudan University, Shanghai 200032, China; 4 Department of Physiology and Pathophysiology, Shanghai Medical College, Fudan University, Shanghai 200032, China; E-Mails: chenying1974@fudan.edu.cn (Y.C.); dyjiong@fudan.edu.cn (Y.D.); yczhu@shmu.edu.cn (Y.Z.)

**Keywords:** urotensin II, apoptosis, cardiomyocyte, cystathionine-γ-lyase (CSE), hydrogen sulfide (H_2_S)

## Abstract

Plasma urotensin II (UII) has been observed to be raised in patients with acute myocardial infarction; suggesting a possible cardiac protective role for this peptide. However, the molecular mechanism is unclear. Here, we treated cultured cardiomyocytes with H_2_O_2_ to induce oxidative stress; observed the effect of UII on H_2_O_2_-induced apoptosis and explored potential mechanisms. UII pretreatment significantly reduced the number of apoptotic cardiomyocytes induced by H_2_O_2_; and it partly abolished the increase of pro-apoptotic protein Bax and the decrease of anti-apoptotic protein Bcl-2 in cardiomyocytes induced by H_2_O_2_. SiRNA targeted to the urotensin II receptor (UT) greatly inhibited these effects. Further analysis revealed that UII increased the production of hydrogen sulfide (H_2_S) and the level of cystathionine-γ-lyase (CSE) by activating the ERK signaling in H_2_O_2_-treated-cardiomyocytes. Si-CSE or ERK inhibitor not only greatly inhibited the increase in CSE level or the phosphorylation of ERK induced by UII but also reversed anti-apoptosis of UII in H_2_O_2_-treated-cadiomyocytes. In conclusion, UII rapidly promoted the phosphorylation of ERK and upregulated CSE level and H_2_S production, which in turn activated ERK signaling to protect cardiomyocytes from apoptosis under oxidative stress. These results suggest that increased plasma UII level may protect cardiomyocytes at the early-phase of acute myocardial infarction in patients.

## 1. Introduction

Urotensin II (UII) is an eleven amino acid cyclic peptide produced from prepro-UII and exhibit full activity in different tissues including the cardiovascular system [1]. UII and its receptor (UT), one of the G protein-coupled receptors (GPCRs), GPR14, are highly expressed in the cardiovascular system and upregulated in pathological conditions such as acute myocardial infarction [2] or chronic hypoxic myocardium [3], suggesting its role in cardiac remodeling. UII has showed a marked mitogenic and proliferative effect on vascular cells [4]. It also affects the apoptosis, hypertrophy, fibrosis, and the inflammation process in various kinds of cells in the cardiovascular system [2,5,6]. However, the effects of UII on cardiovascular function in pathological conditions are still controversial and remain to be explained. Plasma UII has been observed to be raised in patients with acute myocardial infarction and a lower UII response is associated with more severe injury of myocardium [7], indicating a possible cardioprotective role for this peptide. It has been reported that UII can protect the heart against ischemia-reperfusion (I/R) through enhancing coronary flow and reducing cardiac contractility [8]. Reactive oxygen species (ROS) and antioxidant pathway are involved in the protective effect of UII [9]. However, the detailed molecular mechanisms are unclear.

Hydrogen sulfide (H_2_S) emerges as the third physiologically relevant gasotransmitter alongside nitric oxide (NO) and carbon monoxide [10,11]. The production of H_2_S in mammalian systems has been attributed to three principal enzymes: cystathionine-β-synthase (CBS), cystathionine-γ-lyase (CSE) and 3-mercaptopyruvate sulfurtransferase (3-MST). CBS is the predominant source of H_2_S in the central nervous system (CNS). CSE is the major H_2_S-producing enzyme in cardiovascular system, although 3-MST is also expressed in the vascular endothelium [10,12]. It has been reported that H_2_S exerts cytoprotective effects in various models of cardiac injury including I/R injury [13], and suppresses fibrosis by reducing the expression of UII and inhibiting the activation of hepatic stellate cells [14]. But the relationship between UII and H_2_S in injured cardiomyocytes is poorly understood.

Here, we treat cultured cardiomyocytes with H_2_O_2_ to induce oxidative stress, observe the influence of UII on H_2_O_2_-induced apoptosis and detect the production of H_2_S to explore the underlying mechanisms.

## 2. Results and Discussion

### 2.1. UII Prevents Cardiomyocytes from Apoptosis Induced by H_2_O_2_

**Figure 1:**
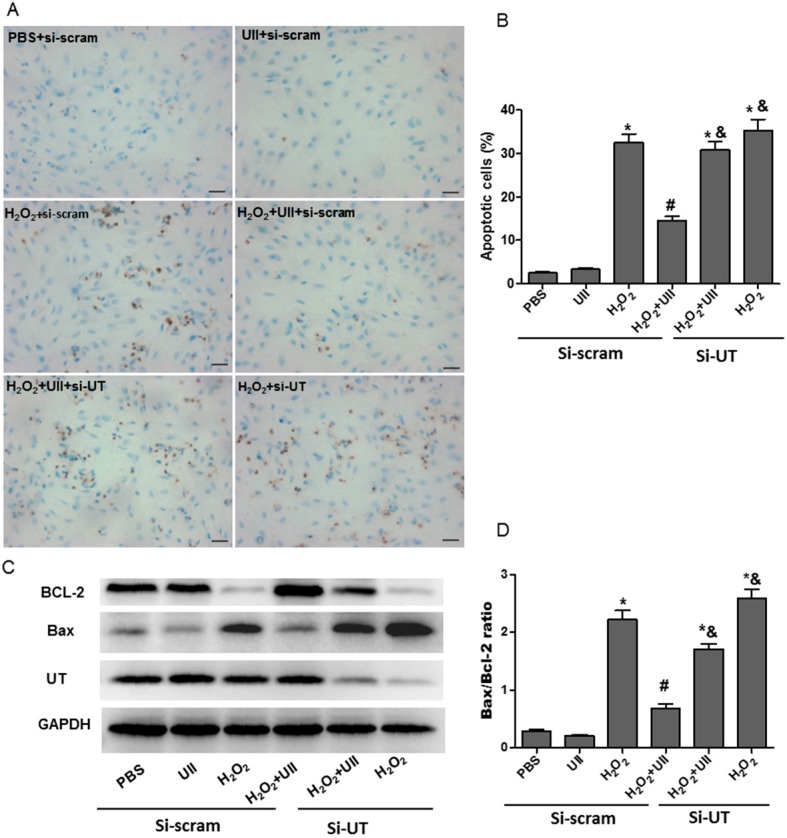
UII prevents cardiomyocytes from apoptosis induced by H_2_O_2_. (**A**) Detection of apoptotic cardiomyocytes (brown nuclei) by TUNEL staining; (**B**) Quantitative analysis of TUNEL positive cells, the data were represented as the percentage of TUNEL positive nuclei; (**C**) Western blot analysis of the level of Bax and Bcl-2 proteins; (**D**) Quantitative analysis of the ratio of Bax/Bcl-2 expression. The cultured cardiomyocytes were transfected with siRNA-UT (si-UT) or siRNA-scramble (as control) for 48 h, then treated with UII (0.1 μM) or PBS for 30 min and exposed to 100 μM H_2_O_2_ for 24 h. si-UT: Cardiomyocytes were transfected with siRNA-UT. Cardiomyocytes were transfected with siRNA-scramble (si-scram) as control. Values are mean ± SEM. * *p* < 0.05 *vs.* PBS group; ^#^ *p* < 0.05 *vs.* H_2_O_2_ group; ^&^ *p* < 0.05 *vs.* H_2_O_2_ + UII group. Bar = 25 μm. All experiments were repeated independently at least three times.

Real-time PCR analysis showed UII mRNA expression was increased significantly in left ventricle tissues at two days after myocardial infarction (Figure S1A). We then explored whether UII can protect cardiomyocytes from apoptosis induced by oxidative stress *in vitro*. According to previous studies [15,16], 100 μM H_2_O_2_ was used to induce oxidative stress-related apoptosis in cardiomyocytes. First, the effect of UII (0.1 µM) [17] on apoptosis induced by H_2_O_2_ was assessed by terminal deoxynucleotidyl transferase-mediated dUTP-biotin nick end labeling assay (TUNEL) in cardiomyocytes. UII pre-treatment significantly reduced the number of TUNEL-positive cardiomyocytes induced by H_2_O_2_. SiRNA targeted UT (si-UT) partly abolished the anti-apoptotic effect of UII (Figure 1A,B). Bax and Bcl-2 have been reported to be a pro-apoptotic protein and an anti-apoptotic protein, respectively [18]. Western blot analysis revealed that H_2_O_2_-treated-cardiomyocytes displayed a significant increase in the level of the pro-apoptotic protein Bax and a decrease in the level of anti-apoptotic protein Bcl-2. But UII greatly prevent the effects, and si-UT significantly suppressed the decrease of Bax and the increase of Bcl-2 induced by UII in H_2_O_2_-treated-cardiomyocytes (Figure 1C,D). These data suggest that UII prevents H_2_O_2_-treated-cardiomyocytes from apoptosis by UT.

UII is a somatostatin-like cyclic peptide synthesized by proteolytic cleavage from prepro-UII that stimulates its receptor (UT) to modulate cardiovascular function in humans and in other animal species [19,20]. The UII/UT receptor-system is expressed widely within the cardiovascular system, and the expression is upregulated in human cardiovascular disease states, including congestive heart failure, essential hypertension, coronary artery disease, type II diabetes, and diabetic nephropathy [21].

The present study showed UII mRNA expression was increased in left ventricle tissues after myocardial infarction. Human UII also causes positive inotropy in human and rat myocardium [22,23], hypertrophic response in cardiomyocytes, and mitogenesis in vascular smooth muscle cells [24] and cardiac fibroblasts [25]. These studies indicate that UII/UT is involved in the development and/or progression of cardiovascular diseases, and the UT receptor is emerging as a promising target for therapeutic intervention [26]. However, a protective effect of UII on the cardiovascular system has been demonstrated recently [27]. In animal models, UII exerts beneficial effects on cardiac injury against ischemic-reperfusion by enhancing coronary flow, reducing cardiac contractility and infarction area [8,28]. In patients with acute myocardial infarction, circulating UII levels are raised which is associated with improved clinical outcomes [7]. We speculate that the contradictory effects of UII may depend on the pathophysiological conditions during cardiovascular diseases. But the molecular mechanisms for the protective effect of UII on cardiomyocytes are unclear.

### 2.2. UII Increases the H_2_S Production by Enhancing the Level of CSE (Cystathionine-γ-Lyase) in Cardiomyocytes Exposed to H_2_O_2_

**Figure 2:**
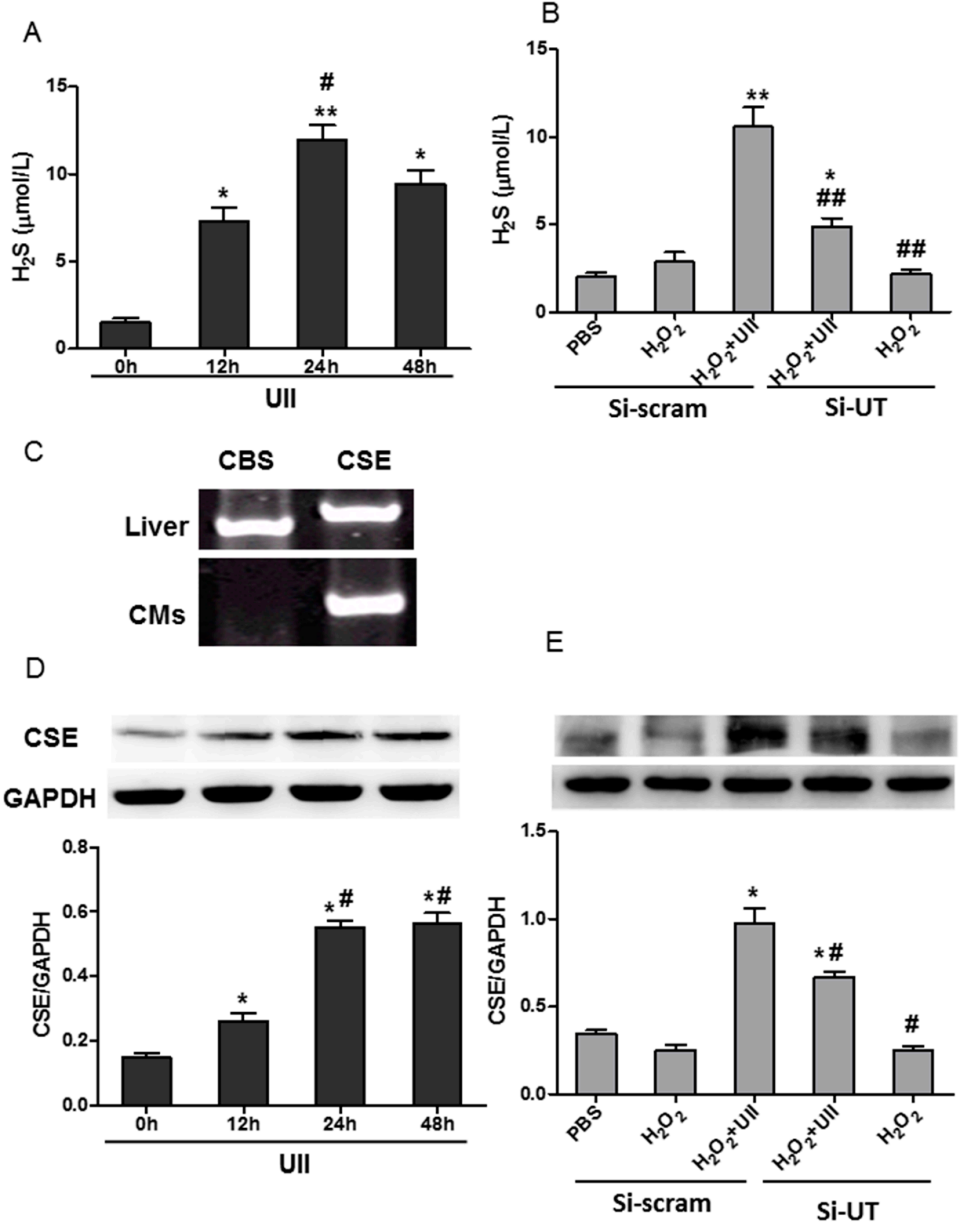
UII increases the production of H_2_S by enhancing the level of CSE in cardiomyocytes exposed to H_2_O_2_. (**A**) H_2_S production was examined in culture supernatant of cardiomyocytes treated with UII at different time points (0, 12, 24, 48 h). Values are mean ± SEM. * *p* < 0.05, ** *p* < 0.01 *vs.* 0 h group; ^#^ *p* < 0.05 *vs.* 12 h group; (**B**) H_2_S production was examined in culture medium of cardiomyocytes transfected with si-UT or siRNA-scramble for 48 h, then treated with UII (0.1 μM) or PBS for 30 min and exposed to 100 μM H_2_O_2_ for 24 h. si-UT: Cardiomyocytes (CMs) were transfected with siRNA-UT, or siRNA-scramble as control. Values are mean ± SEM. * *p* < 0.05, ** *p* < 0.01 *vs.* H_2_O_2_ group; ^##^ *p* < 0.01 *vs.* H_2_O_2_ + UII group; (**C**) RT-PCR analysis of CBS and CSE mRNA expression in cultured cardiomyocytes. The expression of CBS and CSE mRNAs in liver tissue was detected as positive control; (**D**) Western blot analysis of CSE level in cardiomyocytes treated with UII at different time points (0, 12, 24, 48 h). Values are mean ± SEM. * *p* < 0.05 *vs.* 0 h group; ^#^ *p* < 0.05 *vs.* 12 h group; (**E**) Western blot analysis of CSE level in cardiomyocytes transfected with si-UT or siRNA-scramble for 48 h, then treated with UII (0.1 μM) or PBS for 30 min and exposed to 100 μM H_2_O_2_ for 24 h. CMs: Cardiomyocytes. Si-UT: CMs were transfected with siRNA-UT; Si-scram: CMs were transfected with siRNA-scramble as control. Values are mean ± SEM. * *p* < 0.05 *vs.* H_2_O_2_ group; ^#^ *p* < 0.05 *vs.* H_2_O_2_ + UII group. All experiments were repeated independently at least three times.

Recently, H_2_S has been reported to protect against apoptosis in cardiomyocytes stimulated with isoproterenol [29] or oxidative stress [30,31]. We therefore studied the effect of UII on H_2_S production in cardiomyocytes. UII treatment sharply increased the production of H_2_S in cardiomyocytes in time-dependent manner. After 24 h, H_2_S production increased more than three-fold in UII-treated-cardiomyocytes, when compared with control (Figure 2A). H_2_O_2_ has little effect on the production of H_2_S in cardiomyocytes, but UII-pretreatment greatly increased H_2_S production, and si-UT partly abolished the effect of UII in H_2_O_2_-treated-cardiomyocytes (Figure 2B). Endogenous H_2_S is usually produced by CBS or CSE in mammalian systems. CSE is the major H_2_S-producing enzyme in the cardiovascular system [10,12]. Reverse transcription PCR showed that CSE mRNA, not CBS mRNA, was detected in cultured cardiomyocytes (Figure 2C). Western blot analysis revealed that UII significantly upregulated the level of CSE in cardiomyocytes in a time-dependent manner (Figure 2D). At two days after myocardial infarction, UII mRNA level was increased about three-fold compared to that in the sham group, and CSE expression was upregulated accordingly after myocardial infarction (Figure S1B,D). In cultured cardiomyocytes, H_2_O_2_ did not affect the CSE level but UII greatly increased the level in the presence of H_2_O_2_, and the effect was partly abolished by si-UT (Figure 2E). These data revealed that UII promoted H_2_S production by upregulating CSE level in H_2_O_2_-treated-cardiomyocytes.

In recent years, H_2_S, a physiologically relevant gasotransmitter, has been demonstrated to have cytoprotective effects in multiple organ systems including heart. Administration of H_2_S significantly ameliorates myocardial ischaemia–reperfusion injury [32]. Oxidative stress-induced-apoptosis of cardiomyocytes plays critical role in cardiac dysfunction during ischaemia-reperfusion [33]. H_2_S exerts antioxidative effects to protect against apoptosis of cardiomyocytes under oxidative stress through the SIRT1 pathway [31]. In addition, H_2_S may protect the heart or cardiomyocytes against ischemic preconditioning by promoting nitric oxide (NO) release, activating sarcolemmal adenosine triphosphate sensitive K^+^ channel (KATP) [34] or ameliorating intracellular Ca^2+^ handling [35]. UII and its receptor has been reported to be upregulated in ischemic heart tissue [2]. Zhu *et al.* reported that acute myocardial infarction induced the accumulation of CSE in ischemic myocardium [36]. The present data revealed that UII level and CSE expression were upregulated in myocardium after acute myocardial infarction. It indicates that the increased UII may upregulate the expression of CSE under myocardial ischemia. The present data showed that UII enhanced CSE level and H_2_S production which may be involved in the protective role of UII on cardiomyocytes under oxidative stress.

### 2.3. UII Promotes the Activation of ERK in Cultured Cardiomyocytes Exposed to H_2_O_2_

**Figure 3:**
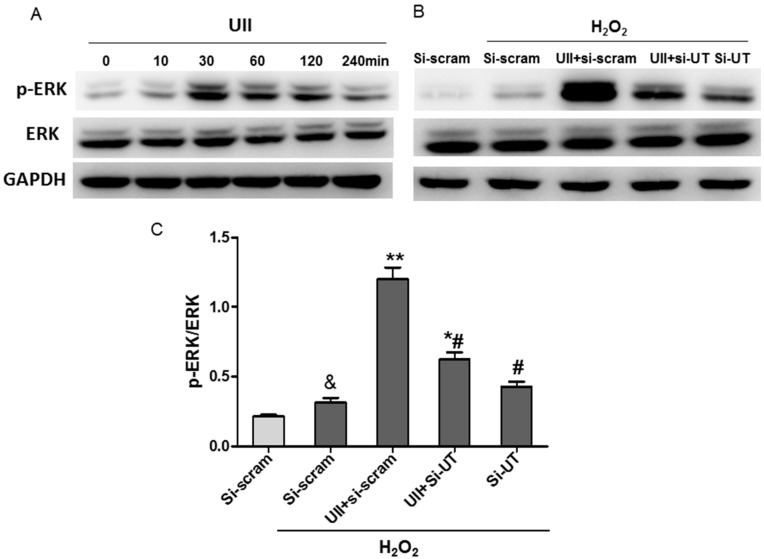
UII promotes the activation of ERK in cultured cardiomyocytes exposed to H_2_O_2_. (**A**) Western blot analysis of p-ERK level in cultured cardiomyocytes treated with UII at different time points (0, 10, 30, 60, 120, 240 min); (**B**) Western blot analysis of p-ERK level in cardiomyocytes; (**C**) Quantitative analysis of p-ERK/ERK level in cultured cardiomyocytes. Cardiomyocytes were transfected with si-UT or siRNA-scramble for 48 h, then treated with UII (0.1 μM) or PBS for 30 min and exposed to 100 μM H_2_O_2_ for 10 min. si-UT: CMs were transfected with siRNA-UT, other CMs were transfected with siRNA-scramble (si-scram) as control. Values are mean ± SEM. ^&^ *p* < 0.05 *vs.* PBS group. * *p* < 0.05, ** *p* < 0.01 *vs.* H_2_O_2_ group; ^#^ *p* < 0.05 *vs.* H_2_O_2_ + UII group. All experiments were repeated independently at least three times.

It has been reported that UII activates ERK to induce cardiomyocytes hypertrophy [21]. ERK is involved in cardioprotection against ischemia [37,38]. The study showed that p-ERK was greatly increased in ventricular tissue at two days after myocardial infarction (Figure S1B,D). To explore whether it is involved in the effect of UII on H_2_S production, we detected the p-ERK level in cardiomyocytes. UII increased p-ERK level in cultured cardiomyocytes in a time-dependent manner (Figure 3A). The level of p-ERK was slightly increased by H_2_O_2_ treatment, but it was greatly upregulated by UII, and the effect was significantly inhibited by si-UT in H_2_O_2_-treated-cardiomyocytes (Figure 3B,C). From our data, we speculate that UII-promoted-activation of ERK may contribute to the increased CSE level and subsequently upregulated endogenous H_2_S production to protect cardiomyocytes against H_2_O_2_. ERK, one of the important mediators of signal transduction from the cell membrane to cytosol or nucleus, is activated by multiple stimuli including ischemia [39,40]. The increased UII level in ischemic heart tissue may be involved in the activation of ERK mediated by the UT pathway. UT (GPR14) belongs to the GPCR family, which is involved in the activation of ERK with multiple mechanisms. G-protein dependent signals probably promotes epidermal growth factor receptor (EGFR) transactivation and then leads to activation of ERK, accounting for the stimulation of Elk1-dependent transcription [41]. In contrast, upon the activation of GPCRs, the binding of cytosolic β-arrestins to ERKs induces the activation of ERK, which may phosphorylate cytosolic kinases involved in multiple regulation [41]. Esposito *et al.* [42] reported that UII induced translocation of β-arrestin 1/2 and EGFR phosphorylation which contributes to the activation of ERK, promoting cell survival and cardioprotection during pressure overload. The activation of integrin-mediated signaling pathways has been reported to be involved in UII-induced phosphorylation of ERK in vascular smooth muscle cells (VSMCs) [43]. In addition, extracellular superoxide dismutase (SOD3) induces ERK activation, which decreases apoptosis in a rat hind limb injury model [40] or in the late stages of the aortopathy [44]. In the present study, UII may activate the ERK pathway through these mechanisms; whether other pathways are involved in the process needs further study.

### 2.4. UII Induces the Reciprocal Regulation of CSE and ERK in Cardiomyocytes Exposed to H_2_O_2_

**Figure 4:**
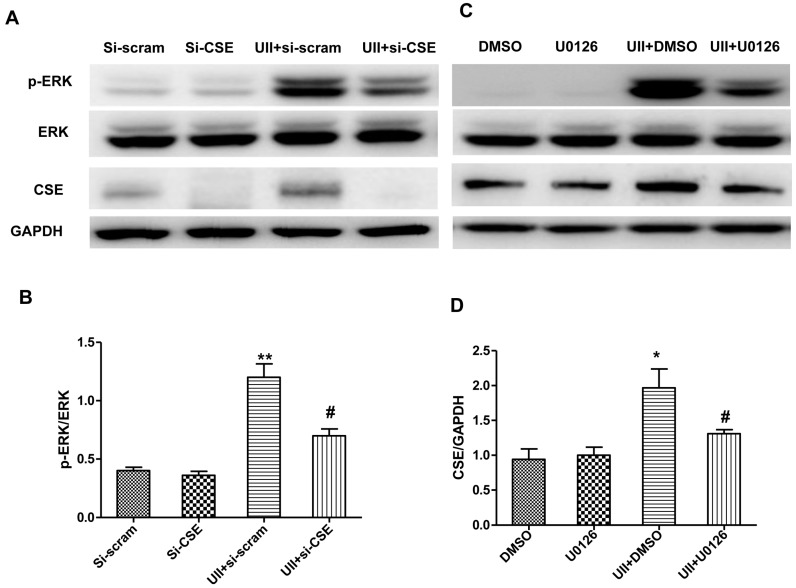
UII induces the reciprocal regulation of CSE and ERK in cardiomyocytes exposed to H_2_O_2_. Representative image of p-ERK, ERK, CSE (**A**) and quantitative analysis of p-ERK/ERK level (**B**) by Western blot analysis in cultured cardiomyocytes. Cardiomyocytes were transfected with si-scramble or si-CSE for 48 h, treated with UII (0.1 μM) or PBS for 30 min, then exposed to 100 μM H_2_O_2_ for 10 min, analyzed for p-ERK and ERK levels, or exposed to 100 μM H_2_O_2_ for 24 h, and analyzed for CSE level. Values are mean ± SEM. ** *p* < 0.01, UII + si-scram *vs.* Si-scram or UII + si-CSE *vs.* Si-CSE. ^#^ *p* < 0.05, UII + si-CSE *vs.* UII + si-scram. Representative image of p-ERK, ERK, CSE (**C**) and quantitative analysis of p-ERK/ERK level (**D**) by Western blot analysis in cultured cardiomyocytes. Cultured cardiomyocytes were treated with DMSO or U0126 with or without UII for 30 min, then exposed to 100 μM H_2_O_2_ for 10 min, analyzed for p-ERK and ERK level, or exposed to 100 μM H_2_O_2_ for 24 h, and analyzed for CSE level. Values are mean ± SEM. * *p* < 0.05, UII + DMSO *vs.* DMSO or UII + U0126 *vs.* UII + DMSO. ^#^ *p* < 0.05, UII + U0126 *vs.* UII + DMSO. All experiments were repeated independently at least three times.

In order to detect the relationship between the increased CSE expression and ERK phosphorylation induced by UII, cardiomyocytes were transfected with siRNA targeted CSE (si-CSE), or treated with the ERK inhibitor, 1,4-diamino-2,3-dicyano-1,4-*bis*(2-aminophenylthio)butadiene (U0126). P-ERK level and CSE levels were detected. Si-CSE effectively mediated knockdown of the expression of CSE in cultured cardiomyocytes, and it greatly inhibited the activation of ERK induced by UII in cardiomyocytes exposed to H_2_O_2_ (Figure 4A,C). ERK, one of the MAPK superfamily, is involved in cellular survival and apoptotic process [45]. Here, we provide evidence that UII-induced-activation of ERK was inhibited by si-CSE in H_2_O_2_-treated-cardiomyocytes. It is reasoned that ERK activation is involved in CSE/H_2_S-mediated-anti-apoptosis in cardiomyocytes under oxidative stress. These findings are in line with a previous study that CSE overexpression mediated protective effects associated with an increased ERK activation in VSMCs [46].

Further analysis showed that the ERK inhibitor, U0126, greatly inhibited not only the phosphorylation of ERK, but also the increased CSE level induced by UII in cardiomyocytes in the presence of H_2_O_2_ (Figure 4B,D). The data revealed that ERK activation may participate in the up-regulation of CSE by UII. CSE expression is regulated by some transcription factors including Elk1 [47]. It has been reported that Elk1 is usually activated by phosphorylation, then interacts with the promoter of CSE and enhances its expression [47] via an ERK-dependent mechanism in liver tissue [48]. Activated ERK, is free to enter the nucleus, and phosphorylates Elk1, contributing to Elk1-dependent transcription [41]. Therefore, UII-induced activation of ERK may account for the increased CSE expression in H_2_O_2_-treated-cardiomyocytes by Elk1 activation. Zhu *et al.* [49] reported that estrogen increased CSE expression and H_2_S generation in the myocardium of ovariectomized rats *in vivo*, but clarification of the mechanisms need further study.

These data suggest that UII induces H_2_S production through increasing CSE levels and the phosphorylation of ERK, which may contribute to the anti-apoptotic effect of UII on cardiomyocytes exposed to H_2_O_2_.

### 2.5. Si-CSE or ERK Inhibitor Partly Abolishes Anti-Apoptotic Effects of UII on H_2_O_2_-Treated Cardiomyocytes

**Figure 5:**
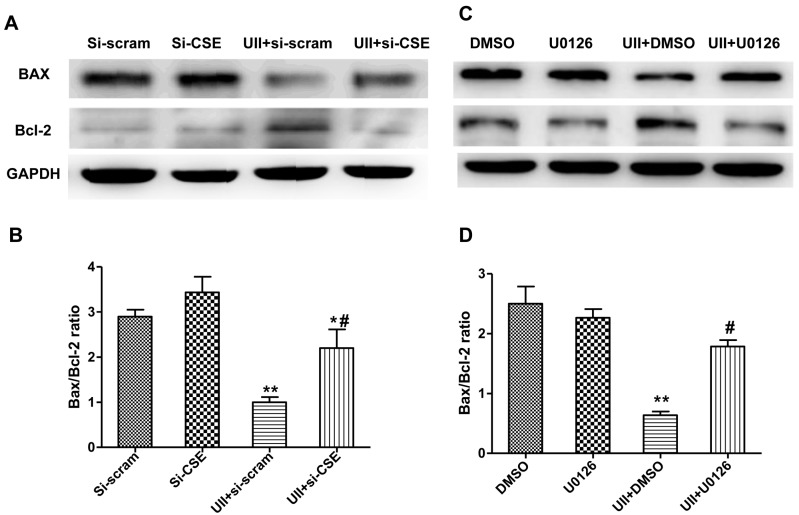
Si-CSE or ERK inhibitor partly abolishes the downregulation of Bax/Bcl-2 induced by UII on H_2_O_2_-treated-cardiomyocytes. Representative image of Bax and Bcl-2 (**A**) and quantitative analysis of Bax/Bcl-2 (**B**) by Western blot analysis in cultured cardiomyocytes transfected with si-scramble or si-CSE for 48 h, treated with UII or PBS for 30 min, then exposed to 100 μM H_2_O_2_ for 24 h. Values are mean ± SEM. * *p* < 0.05, ** *p* < 0.01, UII + si-scram *vs.* Si-scram or UII + si-CSE *vs.* Si-CSE. ^#^ *p* < 0.05 UII + si-CSE *vs.* UII + si-scram. Representative image of Bax and Bcl-2 (**C**) and quantitative analysis of Bax/Bcl-2 (**D**) by Western blot analysis in cultured cardiomyocytes treated with DMSO or U0126 with or without UII for 30 min and exposed to 100 μM H_2_O_2_ for 24 h. Values are mean ± SEM. ** *p* < 0.01, UII + DMSO *vs.* DMSO or UII + U0126 *vs.* UII + DMSO. ^#^ *p* < 0.05, UII + U0126 *vs.* UII + DMSO. All experiments were repeated independently at least three times.

**Figure 6:**
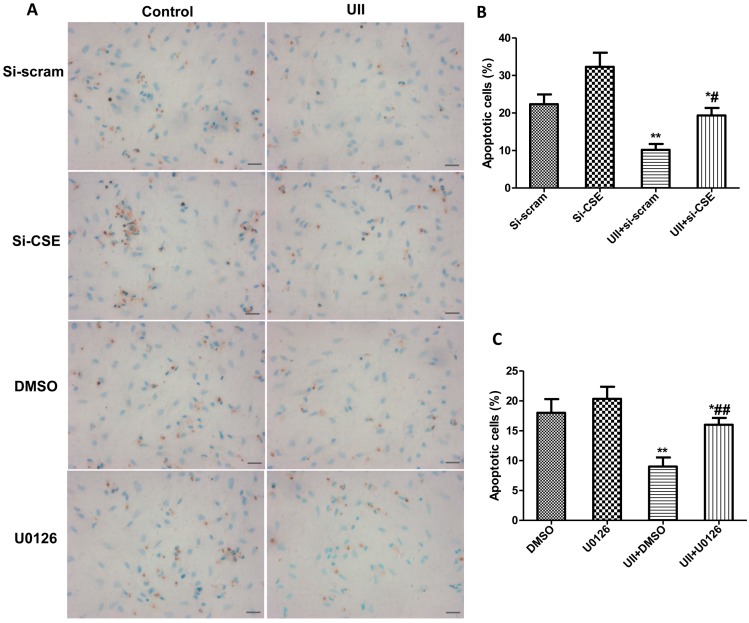
Si-CSE or ERK inhibitor partly abolishes the anti-apoptotic effects of UII on H_2_O_2_-treated-cardiomyocytes. (**A**) Representative images of TUNEL staining in cultured cardiomyocytes. Bar = 25 μm; (**B**) Quantitative analysis of TUNEL staining in cultured cardiomyocytes transfected with si-scramble or si-CSE for 48 h, treated with UII (0.1 μM) or PBS for 30 min, then exposed to 100 μM H_2_O_2_ for 24 h. Values are mean ± SEM. * *p* < 0.05, ** *p* < 0.01, UII + si-scram *vs.* Si-scramb or UII + si-CSE *vs.* Si-CSE. ^#^ *p* < 0.05. UII + si-CSE *vs.* UII + si-scram; (**C**) Quantitative analysis of TUNEL staining in cultured cardiomyocytes treated with DMSO or U0126 with or without UII for 30 min and exposed to 100 μM H_2_O_2_ for 24 h. Values are mean ± SEM. * *p* < 0.05, ** *p* < 0.01, UII *vs.* DMSO or UII + U0126 *vs.* UII + DMSO. ^##^ *p* < 0.01 UII + U0126 *vs.* UII + DMSO. All experiments were repeated independently at least three times.

Cardiomyocytes were transfected with si-CSE, and treated with ERK inhibitor, U0126, respectively. The levels of Bax and Bcl-2 were detected in H_2_O_2_-treated-cardiomyocytes with or without UII pretreatment. Cardiomyocyte apoptosis was analyzed by TUNEL analysis. The results revealed that ERK inhibitor, U0126, or si-CSE reversed the increase in Bcl-2 level and decrease in Bax level induced by UII in cardiomyocytes exposed to H_2_O_2_ (Figure 5A–D). TUNEL analysis showed that UII greatly decreased the number of TUNEL positive cardiomyocytes, but the effect was significantly inhibited by si-CSE or ERK inhibitor in H_2_O_2_-treated-cardiomyocytes (Figure 6A–C).

On the one hand, UII upregulated CSE levels which promoted H_2_S production. The CSE/H_2_S pathway plays an important role in anti-apoptosis against oxidative stress in cardiomyocytes through SIRT or NO signaling as discussed above. On the other hand, UII-induced-activation of ERK may function in another manner to promote survival of cardiomyocytes under oxidative stress. ERK activation has been reported to induce iNOS expression [50] or NF-κB/p65 nucleus translocation [51], which inhibits cell apoptotic processes in other types of cells.

Taken together, these data demonstrate that UII activates ERK rapidly, and the activation of ERK increases CSE level and H_2_S production; in turn, they further upregulate the p-ERK level, which contributes to the anti-apoptotic effect of UII on the cardiomyocytes exposed to H_2_O_2_.

## 3. Experimental Section

### 3.1. Primary Culture of Neonatal Rat Cardiomyocytes

Primary culture of neonatal cardiomyocytes (CMs) was prepared from 1–3 days old Sprague-Dawley (SD) rats by the trypsin digestion method, as previously described [15]. Briefly, rat ventricles were minced into pieces and subjected to 0.125% trypsin digestion in Hank's Balanced Salt Solution (HBSS). After 2 h of cell attachment to remove non-cardiomyocytes, CMs were collected and maintained for 48 h in DMEM/F12 containing 10% FBS with antibiotics. A supplement of 0.1 mmol/mL 5-bromo-2ʹ-deoxyuridine (Brdu, Sigma-Aldrich, St. Louis, MO, USA) was added to cultured medium to inhibit non-cardiomyocytes. Then the cells were deprived of serum for 24 h prior to experiments.

### 3.2. Apoptotic Cell Analysis by TUNEL Labeling

TUNEL labeling was performed according to the manufacturer’s protocol (*In situ* Cell Death Detection kit, Merck Inc., Billerica, MA, USA). The slides were incubated with 50 μL TUNEL reaction mixture containing TdT for 60 min at 37 °C, and then incubated with diaminobenzidine (DAB) substrate solution for 10 min. The apoptotic cells were expressed as a percentage of the TUNEL positive nuclei in all the nuclei of detected cell samples [52]. Five fields per cell sample were evaluated and averaged. At least 3 cell samples of each group were analyzed. 

### 3.3. H_2_S Measurement

The H_2_S level in culture supernatant was indicated by sulfide using the method previously described with some modifications [53]. Briefly, cells were cultured in phenol red-free DMEM supplemented with 10% FBS, and stimulated with 100 μM H_2_O_2_ with or without UII (0.1 μM, Sigma) pretreatment for the time periods as indicated. After that, 500 μL culture supernatants were collected and added into a test tube containing 250 μL 1% zinc acetate, followed by the addition with 133 μL of 20 mM *N*,*N*-dimethyl-*p*-phenylenediamine sulfate in 7.2 M HCl and 133 μL of 30 mM FeCl_3_ in 1.2 M HCl. Subsequently, the mixtures were incubated at room temperature for 20 min. Afterwards, the protein in the culture supernatant was removed by adding 250 μL 10% trichloroacetic acid; the absorbance of the resulting solution was detected at 670 nm by a microplate reader (Infinite M200, Tecan, Seestrasse, Switzerland). The H_2_S concentration was assessed by a standard curve of NaHS.

### 3.4. Reverse Transcription PCR (RT-PCR)

Total RNA was extracted from cardiomyocytes or liver tissue of male SD rats (200–250 g) with Trizol (Invitrogen, Carlsbad, CA, USA) following the manufacturer’s instructions. Total RNA (1 μg) of each sample was reversely transcribed into cDNA using a cDNA synthesis kit (Fermentas, Burlington, ON, Canada). An equal volume of the resulting cDNA product was amplified using a PCR Master Mix kit (Fermentas, USA) with primers (Shenggong, Shanghai, China) as follows: rat CSE (NM_017074.1), forward: 5′-TCCGGATGGAGAAACACTTC-3′; reverse: 5′-CACCAGCATGTCCACCTTC-3′; CBS (NM_012522.2) forward: 5′-GCCAACTTCTGGCAACAC-3′, reverse: 5′-CACCAGCATGTCCACCTTC-3′) and GAPDH (forward 5′-AACAAGCAACTGTCCCTGAGC-3′, reverse: 5′-GTAGACAGAAGGTGGCACAGA-3′). The PCR conditions included denaturation at 94 °C for 3 min, followed by 30 repeated cycles of 95 °C for 30 s, 55 °C for 45 s and 72 °C for 45 s, and extension at 72 °C for 7 min. PCR products (372 bp for CSE, 307 bp for CBS) were separated in 2% agarose gels. The optical band densities were analyzed with Image software program (FR-200A, CapitalBio, Beijing, China).

### 3.5. SiRNA and in Vitro Transfection

SiRNAs for CSE or UT were obtained from Shanghai GenePharma (Shanghai, China), and three different sequences targeted to rat CSE or UT were screened for the efficiency of knockdown CSE or UT in cardiomyocytes, respectively. Lipofectamin 2000 (Sigma-Aldrich, St. Louis, MO, USA) and siRNAs were mixed and kept still for 20 min at room temperature, and then the mixtures were added to cardiomyocytes cultured in 6-well plates for transfection. The scramble sequences were also transfected as controls.

### 3.6. Western Blot Analysis

Cultured cardiomyocytes were transfected with si-scramble or si-CSE for 48 h, treated with UII (0.1 μM) or PBS for 30 min, then exposed to 100 μM H_2_O_2_ for 10 min or 24 h, or cultured cardiomyocytes were treated with DMSO or U0126 (Sigma-Aldrich, St. Louis, MO, USA ) with or without UII for 30 min and exposed to 100 μM H_2_O_2_ for 10 min or 24 h. Protein was extracted from these cardiomyocytes with lysis buffer according to previous study [16]. The protein samples were separated in 12% SDS-PAGE, transferred to polyvinylidene difluoride membranes (Gelman-Pall, Ann Arbor, MI, USA), and then incubated overnight at 4 °C with primary antibodies: GPR14 (1:500 Santa Cruz Biotechnology, Dallas, TX, USA), CSE (1:1000), phosphorylated ERK (1:5000), ERK(1:5000), Bcl-2 (1:1000) (all Cell Signaling Technology, Danvers, MA, USA) and Bax (6A7) (1:1000) (Abcam, Cambridge, MA, USA), followed by incubation with horseradish peroxidase (HRP)-conjugated secondary antibodies (1:1000) for 1 h at room temperature. And the results were developed by Pro-Light chemiluminescent detection kit (Tiangen Biotech Inc., Beijing, China) with LAS-3000 imaging system (FUJIFILM Inc., Tokyo, Japan).

### 3.7. Myocardial Infarction Model of Mouse and Real-Time Polymerase Chain Reaction (RT-PCR)

Please refer to “Supplementary Methods”.

### 3.8. Statistical Analysis

All the data were presented as mean ± SEM and were analyzed using One-Way ANOVA tests followed by Least-Significance-Difference (LSD) method for multiple means comparison or using Independent-Samples *t*-tests for two means comparison. A two-tailed *p*-value of <0.05 was considered statistically significant. All statistical analysis was performed by Prism 6.0 (GraphPad Software. Inc, San Diego, CA, USA).

## 4. Conclusions

Taken together, UII rapidly promoted the phosphorylation of ERK, and increased CSE level and H_2_S production, which in turn enhanced the p-ERK level and contributed to the anti-apoptosis of UII on cardiomyocytes under oxidative stress. This mechanism may be critical for the protective effect of UII on cardiac injury against ischemia reperfusion.

## Supplementary Materials

Supplementary materials can be found at http://www.mdpi.com/1422-0067/16/06/12482/s1.

## References

[1] Russell F.D., Meyers D., Galbraith A.J., Bett N., Toth I., Kearns P., Molenaar P. (2003). Elevated plasma levels of human urotensin-II immunoreactivity in congestive heart failure. Am. J. Physiol..

[2] Tzanidis A., Hannan R.D., Thomas W.G., Onan D., Autelitano D.J., See F., Kelly D.J., Gilbert R.E., Krum H. (2003). Direct actions of urotensin II on the heart: Implications for cardiac fibrosis and hypertrophy. Circ. Res..

[3] Zhang Y., Li J., Cao J., Chen J., Yang J., Zhang Z., Du J., Tang C. (2002). Effect of chronic hypoxia on contents of urotensin II and its functional receptors in rat myocardium. Heart Vessel..

[4] Djordjevic T., BelAiba R.S., Bonello S., Pfeilschifter J., Hess J., Gorlach A. (2005). Human urotensin II is a novel activator of NADPH oxidase in human pulmonary artery smooth muscle cells. Arterioscler. Thromb. Vasc. Biol..

[5] Johns D.G., Ao Z., Naselsky D., Herold C.L., Maniscalco K., Sarov-Blat L., Steplewski K., Aiyar N., Douglas S.A. (2004). Urotensin-II-mediated cardiomyocyte hypertrophy: Effect of receptor antagonism and role of inflammatory mediators. Naunyn Schmiedebergs Arch. Pharmacol..

[6] Russell F.D. (2004). Emerging roles of urotensin-II in cardiovascular disease. Pharmacol. Ther..

[7] Khan S.Q., Bhandari S.S., Quinn P., Davies J.E., Ng L.L. (2007). Urotensin II is raised in acute myocardial infarction and low levels predict risk of adverse clinical outcome in humans. Int. J. Cardiol..

[8] Prosser H.C., Forster M.E., Richards A.M., Pemberton C.J. (2008). Urotensin II and urotensin II-related peptide (URP) in cardiac ischemia-reperfusion injury. Peptides.

[9] Gao S., Oh Y.B., Park B.M., Park W.H., Kim S.H. (2012). Urotensin II protects ischemic reperfusion injury of hearts through ROS and antioxidant pathway. Peptides.

[10] Wang R. (2002). Two’s company, three’s a crowd: Can H_2_S be the third endogenous gaseous transmitter?. FASEB J..

[11] Wang R. (2012). Physiological implications of hydrogen sulfide: A whiff exploration that blossomed. Physiol. Rev..

[12] Shibuya N., Mikami Y., Kimura Y., Nagahara N., Kimura H. (2009). Vascular endothelium expresses 3-mercaptopyruvate sulfurtransferase and produces hydrogen sulfide. J. Biochem..

[13] Peake B.F., Nicholson C.K., Lambert J.P., Hood R.L., Amin H., Amin S., Calvert J.W. (2013). Hydrogen sulfide preconditions the db/db diabetic mouse heart against ischemia-reperfusion injury by activating Nrf2 signaling in an Erk-dependent manner. Am. J. Physiol. Heart Circ. Physiol..

[14] Liu Y., Li Y., Yang W., Cao G. (2013). H_2_S inhibits the activation of hepatic stellate cells and downregulates the expression of urotensin II. Hepatol. Res..

[15] Ma H., Gong H., Chen Z., Liang Y., Yuan J., Zhang G., Wu J., Ye Y., Yang C., Nakai A. (2012). Association of Stat3 with HSF1 plays a critical role in G-CSF-induced cardio-protection against ischemia/reperfusion injury. J. Mol. Cell Cardiol..

[16] Shan P., Pu J., Yuan A., Shen L., Shen L., Chai D., He B. (2008). RXR agonists inhibit oxidative stress-induced apoptosis in H9c2 rat ventricular cells. Biochem. Biophys. Res. Commun..

[17] Zou Y., Nagai R., Yamazaki T. (2001). Urotensin II induces hypertrophic responses in cultured cardiomyocytes from neonatal rats. FEBS Lett..

[18] Cheng E.H., Wei M.C., Weiler S., Flavell R.A., Mak T.W., Lindsten T., Korsmeyer S.J. (2001). BCL-2, BCL-X(L) sequester BH3 domain-only molecules preventing BAX- and BAK-mediated mitochondrial apoptosis. Mol. Cell.

[19] Coulouarn Y., Lihrmann I., Jegou S., Anouar Y., Tostivint H., Beauvillain J.C., Conlon J.M., Bern H.A., Vaudry H. (1998). Cloning of the cDNA encoding the urotensin II precursor in frog and human reveals intense expression of the urotensin II gene in motoneurons of the spinal cord. Proc. Natl. Acad. Sci. USA.

[20] Ames R.S., Sarau H.M., Chambers J.K., Willette R.N., Aiyar N.V., Romanic A.M., Louden C.S., Foley J.J., Sauermelch C.F., Coatney R.W. (1999). Human urotensin-II is a potent vasoconstrictor and agonist for the orphan receptor GPR14. Nature.

[21] Zhu Y.C., Zhu Y.Z., Moore P.K. (2006). The role of urotensin II in cardiovascular and renal physiology and diseases. Br. J. Pharmacol..

[22] Gong H., Wang Y.X., Zhu Y.Z., Wang W.W., Wang M.J., Yao T., Zhu Y.C. (2004). Cellular distribution of GPR14 and the positive inotropic role of urotensin II in the myocardium in adult rat. J. Appl. Physiol..

[23] Russell F.D., Molenaar P., O’Brien D.M. (2001). Cardiostimulant effects of urotensin-II in human heart in vitro. Br. J. Pharmacol..

[24] Sauzeau V., Le Mellionnec E., Bertoglio J., Scalbert E., Pacaud P., Loirand G. (2001). Human urotensin II-induced contraction and arterial smooth muscle cell proliferation are mediated by RhoA and Rho-kinase. Circ. Res..

[25] Chen Y.L., Liu J.C., Loh S.H., Chen C.H., Hong C.Y., Chen J.J., Cheng T.H. (2008). Involvement of reactive oxygen species in urotensin II-induced proliferation of cardiac fibroblasts. Eur. J. Pharmacol..

[26] Ross B., McKendy K., Giaid A. (2010). Role of urotensin II in health and disease. Am. J. Physiol. Regul. Integr. Comp. Physiol..

[27] Russell F.D. (2008). Urotensin II in cardiovascular regulation. Vasc. Health Risk Manag..

[28] Gao Y., Liu C.Y., Zhang X.J., Gao J., Yang C.Y. (2008). Circulating endothelial cells as potential markers of atherosclerosis. Can. J. Neurol. Sci..

[29] Lu F., Xing J., Zhang X., Dong S., Zhao Y., Wang L., Li H., Yang F., Xu C., Zhang W. (2013). Exogenous hydrogen sulfide prevents cardiomyocyte apoptosis from cardiac hypertrophy induced by isoproterenol. Mol. Cell. Biochem..

[30] Mishra P.K., Tyagi N., Sen U., Givvimani S., Tyagi S.C. (2010). H_2_S ameliorates oxidative and proteolytic stresses and protects the heart against adverse remodeling in chronic heart failure. Am. J. Physiol. Heart Circ. Physiol..

[31] Wu D., Hu Q., Liu X., Pan L., Xiong Q., Zhu Y.Z. (2015). Hydrogen sulfide protects against apoptosis under oxidative stress through SIRT1 pathway in H9c2 cardiomyocytes. Nitric Oxide.

[32] King A.L., Lefer D.J. (2011). Cytoprotective actions of hydrogen sulfide in ischaemia-reperfusion injury. Exp. Physiol..

[33] Pu J., Yuan A., Shan P., Gao E., Wang X., Wang Y., Lau W.B., Koch W., Ma X.L., He B. (2013). Cardiomyocyte-expressed farnesoid-X-receptor is a novel apoptosis mediator and contributes to myocardial ischaemia/reperfusion injury. Eur. Heart J..

[34] Pan T.T., Feng Z.N., Lee S.W., Moore P.K., Bian J.S. (2006). Endogenous hydrogen sulfide contributes to the cardioprotection by metabolic inhibition preconditioning in the rat ventricular myocytes. J. Mol. Cell. Cardiol..

[35] Pan T.T., Neo K.L., Hu L.F., Yong Q.C., Bian J.S. (2008). H_2_S preconditioning-induced PKC activation regulates intracellular calcium handling in rat cardiomyocytes. Am. J. Physiol. Cell Physiol..

[36] Zhu Y.Z., Wang Z.J., Ho P., Loke Y.Y., Zhu Y.C., Huang S.H., Tan C.S., Whiteman M., Lu J., Moore P.K. (2007). Hydrogen sulfide and its possible roles in myocardial ischemia in experimental rats. J. Appl. Physiol..

[37] Wu X., Xu T., Li D., Zhu S., Chen Q., Hu W., Pan D., Zhu H., Sun H. (2013). ERK/PP1a/PLB/SERCA2a and JNK pathways are involved in luteolin-mediated protection of rat hearts and cardiomyocytes following ischemia/reperfusion. PLoS ONE.

[38] Yin Y., Guan Y., Duan J., Wei G., Zhu Y., Quan W., Guo C., Zhou D., Wang Y., Xi M., Wen A. (2013). Cardioprotective effect of Danshensu against myocardial ischemia/reperfusion injury and inhibits apoptosis of H9c2 cardiomyocytes via Akt and ERK1/2 phosphorylation. Eur. J. Pharmacol..

[39] Shimizu N., Yoshiyama M., Omura T., Hanatani A., Kim S., Takeuchi K., Iwao H., Yoshikawa J. (1998). Activation of mitogen-activated protein kinases and activator protein-1 in myocardial infarction in rats. Cardiovasc. Res..

[40] Laatikainen L.E., Incoronato M., Castellone M.D., Laurila J.P., Santoro M., Laukkanen M.O. (2011). SOD3 decreases ischemic injury derived apoptosis through phosphorylation of Erk1/2, Akt, and FoxO3a. PLoS ONE.

[41] Luttrell L.M. (2002). Activation and targeting of mitogen-activated protein kinases by G-protein-coupled receptors. Can. J. Physiol. Pharmacol..

[42] Esposito G., Perrino C., Cannavo A., Schiattarella G.G., Borgia F., Sannino A., Pironti G., Gargiulo G., di Serafino L., Franzone A. (2011). EGFR trans-activation by urotensin II receptor is mediated by beta-arrestin recruitment and confers cardioprotection in pressure overload-induced cardiac hypertrophy. Basic Res. Cardiol..

[43] Tamura K., Okazaki M., Tamura M., Isozumi K., Tasaki H., Nakashima Y. (2003). Urotensin II-induced activation of extracellular signal-regulated kinase in cultured vascular smooth muscle cells: Involvement of cell adhesion-mediated integrin signaling. Life Sci..

[44] Arcucci A., Ruocco M.R., Albano F., Granato G., Romano V., Corso G., Bancone C., de Vendittis E., della Corte A., Montagnani S. (2014). Analysis of extracellular superoxide dismutase and Akt in ascending aortic aneurysm with tricuspid or bicuspid aortic valve. Eur. J. Histochem..

[45] Xia Z., Dickens M., Raingeaud J., Davis R.J., Greenberg M.E. (1995). Opposing effects of ERK and JNK-p38 MAP kinases on apoptosis. Science.

[46] Yang G., Wu L., Wang R. (2006). Pro-apoptotic effect of endogenous H_2_S on human aorta smooth muscle cells. FASEB J..

[47] Taniguchi S., Kimura T., Umeki T., Kimura Y., Kimura H., Ishii I., Itoh N., Naito Y., Yamamoto H., Niki I. (2012). Protein phosphorylation involved in the gene expression of the hydrogen sulphide producing enzyme cystathionine gamma-lyase in the pancreatic β-cell. Mol. Cell. Endocrinol..

[48] Guha M., O’Connell M.A., Pawlinski R., Hollis A., McGovern P., Yan S.F., Stern D., Mackman N. (2001). Lipopolysaccharide activation of the MEK-ERK1/2 pathway in human monocytic cells mediates tissue factor and tumor necrosis factor alpha expression by inducing Elk-1 phosphorylation and Egr-1 expression. Blood.

[49] Zhu X., Tang Z., Cong B., Du J., Wang C., Wang L., Ni X., Lu J. (2013). Estrogens increase cystathionine-gamma-lyase expression and decrease inflammation and oxidative stress in the myocardium of ovariectomized rats. Menopause.

[50] Kim S.J., Chun J.Y., Kim M.S. (2000). Insulin stimulates production of nitric oxide via ERK in osteoblast cells. Biochem. Biophys. Res. Commun..

[51] Shi M., He X., Wei W., Wang J., Zhang T., Shen X. (2015). Tenascin-C induces resistance to apoptosis in pancreatic cancer cell through activation of ERK/NF-κB pathway. Apoptosis.

[52] Pu J., Mintz G.S., Biro S., Lee J.B., Sum S.T., Madden S.P., Burke A.P., Zhang P., He B., Goldstein J.A. (2014). Insights into echo-attenuated plaques, echolucent plaques, and plaques with spotty calcification: novel findings from comparisons among intravascular ultrasound, near-infrared spectroscopy, and pathological histology in 2294 human coronary artery segments. J. Am. Coll. Cardiol..

[53] Cai W.J., Wang M.J., Moore P.K., Jin H.M., Yao T., Zhu Y.C. (2007). The novel proangiogenic effect of hydrogen sulfide is dependent on Akt phosphorylation. Cardiovasc. Res..

