# Supplementary material for: Urotensin II Protects Cardiomyocytes from Apoptosis Induced by Oxidative Stress through the CSE/H2S Pathway
Source: Int J Mol Sci. 2015 Jun 3;16(6):12482–98. doi: 10.3390/ijms160612482 (PMC4490456; doi:10.3390/ijms160612482)
Supplement: Supplementary file 1 [file ijms-16-12482-s001.pdf]

Supplementary Information

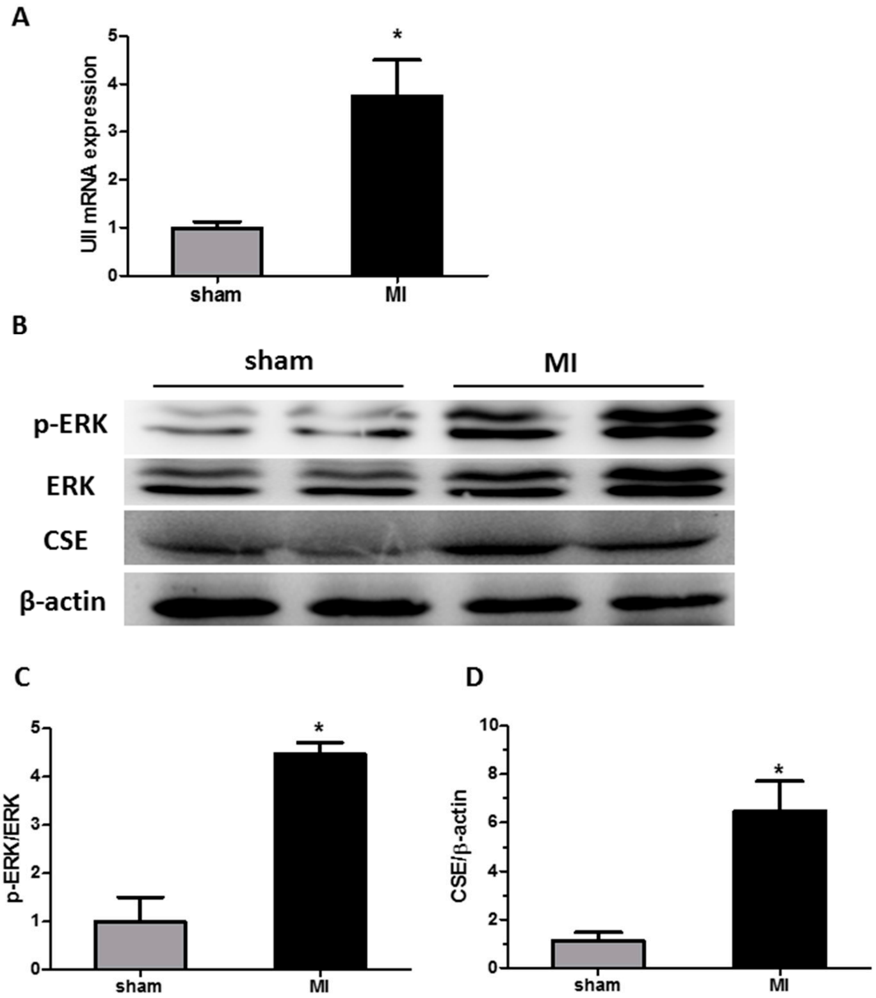

Figure S1. The expressions of UII mRNA, p-ERK and CSE were analyzed in left ventricle tissues at 2 days after myocardial infarction. (**A**) Real-time PCR analysis of UII mRNA level; (**B**) The representative picture of p-ERK and CSE expression by western blot analysis; (**C**) Quantitative analysis of p-ERK expression by western blot analysis; (**D**) Quantitative analysis of CSE expression by western blot analysis. Sham: sham operation (n = 4). MI: myocardial infarction (n = 4). * $p < 0.05$ *vs.* sham group.

Supplementary Methods

Myocardial Infarction Model of Mouse

Myocardial infarction model of mice was established as previous study [2]. C57BL/6 mice (8–10 weeks) were subjected to occlusion by an 8-0 silk on the top of the left anterior descending artery or sham operation after anesthetized with ketamine and artificially ventilated with a respirator. The ischemia injury was identified by discoloration of left ventricle and electrocardiogram. At 2 days after surgery, the whole hearts were excised, and snap frozen over liquid nitrogen for subsequent western blot analysis.

Real-Time Polymerase Chain Reaction (RT-PCR)

Total RNAs were extracted from heart tissue as described in "Experimental Section", Real-Time PCR was performed with Power SYBR Green PCR Master Mix (TaKaRa, Otsu, Japan) and analyzed by the iCycler System (Bio-Rad, Hercules, CA, USA). The primers were used for real-time PCR: UII, forward primer: AACATAAGCAACACGGGGCT; Reverse primer: AAGGCGTCGTTAGCGTGATT. GAPDH, forward primer: GGTGAAGGTCGGTGTGAACGGATT, Reverse primer: AATGCCAAA GTTGTCATGGATGACC.

Relative levels of UII mRNA expression were normalized to the GAPDH mRNA expression by using the comparative C_t method.
